# A Biomimetic Optical Cardiac Fibrosis-on-a-Chip for High-Throughput Anti-Fibrotic Drug Screening

**DOI:** 10.34133/research.0471

**Published:** 2024-09-12

**Authors:** Yixuan Shang, Dongyu Xu, Lingyu Sun, Yuanjin Zhao, Lingyun Sun

**Affiliations:** ^1^ Department of Rheumatology and Immunology, Nanjing Drum Tower Hospital, Affiliated Hospital of Medical School, Nanjing University, Nanjing 210008, China.; ^2^ State Key Laboratory of Bioelectronics, School of Biological Science and Medical Engineering, Southeast University, Nanjing 210096, China.; ^3^ Department of Rheumatology and Immunology, The First Affiliated Hospital of Anhui Medical University, Hefei 230022, China.

## Abstract

Cardiac fibrosis has emerged as the primary cause of morbidity, disability, and even mortality in numerous nations. In light of the advancements in precision medicine strategies, substantial attention has been directed toward the development of a practical and precise drug screening platform customized for individual patients. In this study, we introduce a biomimetic cardiac fibrosis-on-a-chip incorporating structural color hydrogels (SCHs) to enable optical high-throughput drug screening. By cocultivating a substantial proportion of cardiac fibroblasts (CFBs) with cardiomyocytes on the SCH, this biomimetic fibrotic microtissue successfully replicates the structural components and biomechanical properties associated with cardiac fibrosis. More importantly, the structural color shift observed in the SCH can be indicative of cardiac contraction and relaxation, making it a valuable tool for evaluating fibrosis progression. By incorporating such fibrotic microtissue into a microfluidic gradient chip, we develop a biomimetic optical cardiac fibrosis-on-a-chip platform that accurately and efficiently screens potential anti-fibrotic drugs. These characteristics suggest that this microphysiological platform possesses the capability to establish a preclinical framework for screening cardiac drugs, and may even contribute to the advancement of precision medicine.

## Introduction

Cardiac fibrosis is a prevalent pathophysiological phenomenon observed in the majority of cardiovascular diseases, which stand as the primary contributors to morbidity, disability, and even mortality in numerous countries [1–3]. Considering the pathogenic processes underlying cardiac fibrosis, extensive research efforts have been focused on exploring diverse compounds, notably those that inhibit angiotensin II (Ang-II), modulate inflammation, or regulate pathways related to transforming growth factor-β (TGF-β) [4–6]. These investigations have yielded promising results with potential clinical applications. Nonetheless, variability in treatment efficacy arises from differences in drug tolerance among patients, highlighting the dependence of traditional clinical approaches on subjective evaluations by healthcare professionals. Once inappropriate medication is administered, it often leads to prolonged treatment periods and even severe and harmful side effects. Therefore, an effective and precise drug screening platform for patients is eagerly anticipated.

**Fig. 1.**
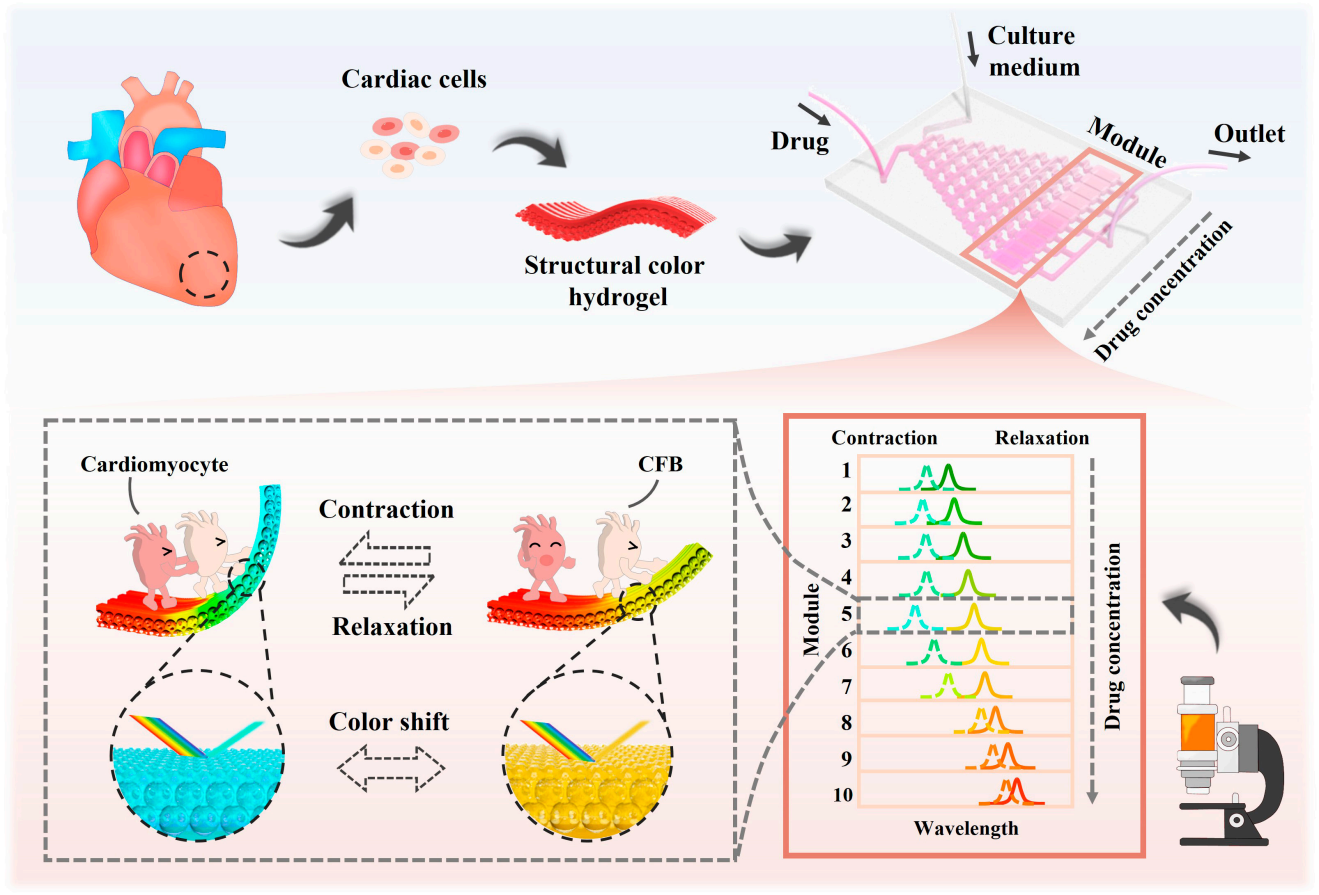
Concept map of the biomimetic optical cardiac fibrosis-on-a-chip for evaluating anti-fibrotic drug therapy.

In this paper, we proposed a biomimetic optical cardiac fibrosis-on-a-chip based on structural color hydrogel (SCH) for evaluating anti-fibrotic drug therapy, as schemed in Fig. 1. Organ-on-chips have been regarded as valuable bionic systems for investigating human diseases [7–12]. Diverse heart disease chips with similar extracellular matrix (ECM) composition and cellular functionality to those in vivo have been established, greatly facilitating a deeper comprehension of cardiac diseases and cardiovascular drug discovery [13]. Especially, owing to the biomimetic structure and composition design of structural color materials, cardiac chips integrated with these materials have garnered considerable attention for their noninvasive sensing and visualization of mechanical signals [14–16]. Leveraging these advantages, such cardiac chips have found wide application in multidimensional detection and intelligent drug screening fields [13,17]. Despite the advancements, there has been limited research on high-throughput screening of anti-fibrotic drugs in such cardiac chips.

Herein, we constructed an optical high-throughput drug screening system for assessing fibrosis reversal efficacy using a combination of microfluidic gradient design and structural color sensing modules. To mimic fibrotic microtissues, a high proportion of cardiac fibroblasts (CFBs) were cocultured with cardiomyocytes on an SCH with biomimetic topology. After cocultivation, our biomimetic fibrotic microtissue presented fibrotic characteristic hallmarks, for instance, fibrotic remodeling, impaired contractile force, and aberrant calcium handling. Because of synchronized deformation and corresponding structural color shifts induced by myocardial beating, the SCH with cultured cardiac cells displayed comprehensive visual criteria for assessing fibrosis progression. By further integrating these fibrotic microtissues into an incorporated microfluidic gradient chip, we obtained a biomimetic fibrotic chip featuring microphysiological visuals, suitable for high-throughput drug screening. In proof-of-principle tests, 5 promising anti-fibrotic drugs were selected and their optimal dosage and overall effectiveness were systematically evaluated. These features underscore the potential of employing multi-parameter analysis and concentration gradient channels to advance the development of biomimetic fibrotic chips, thereby expanding their utility in drug screening applications.

## Results and Discussion

**Fig. 2.**
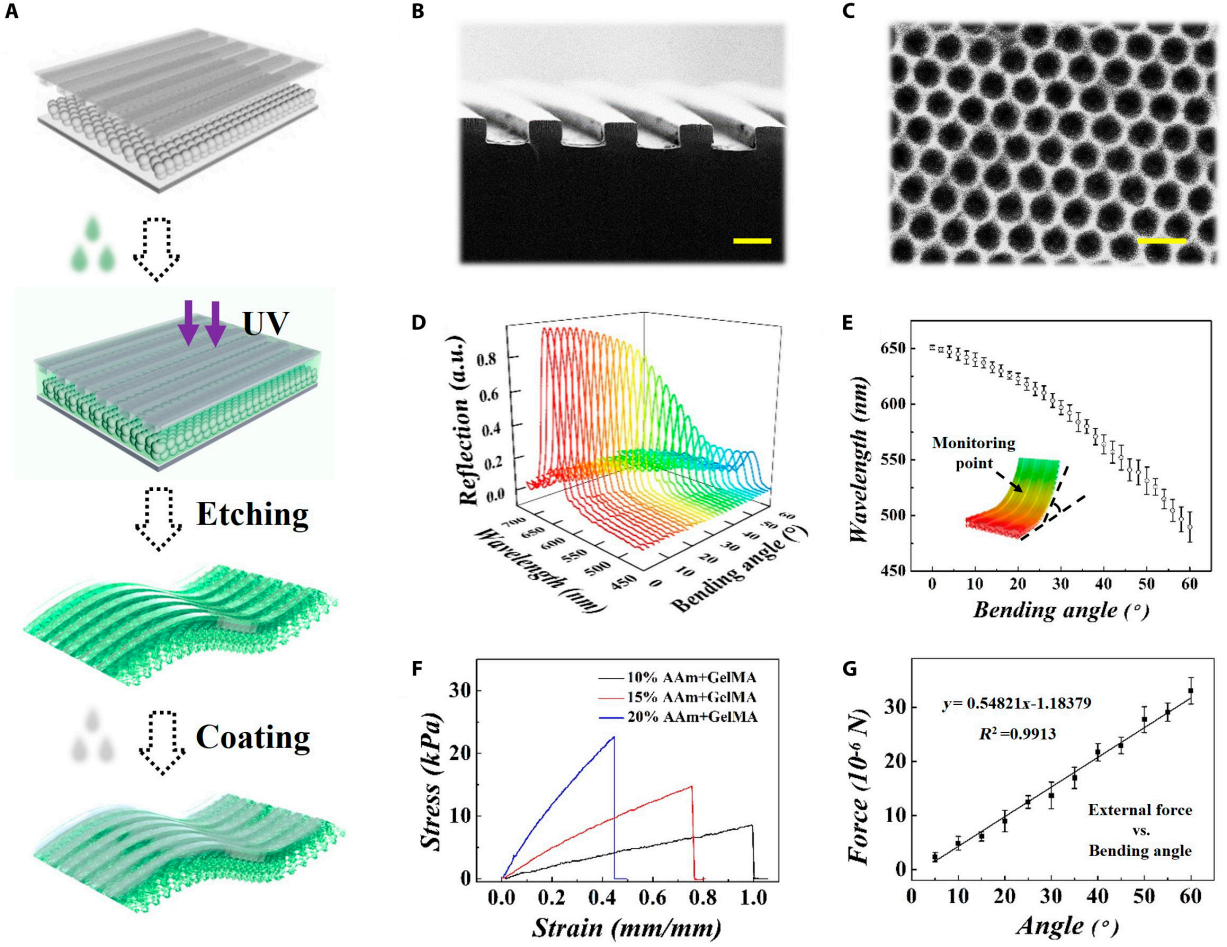
(A) Schematic illustration of the fabrication process of SCH. (B and C) Scanning electron microscope (SEM) images showing the microgroove patterns on the upside (B) and inverse opal nanostructure on the bottom side (C) of SCH. (D and E) Reflection spectra of SCH with incremental bending angle. (F) Stress–strain curves of SCHs fabricated by diverse concentrations of AAm. (G) Relationship of external forces and resulting bending angles of SCH with 15% AAm. Scale bars, 20 μm (B) and 0.5 μm (C).

In a typical experiment, the SCHs with surface topology were developed by template replicating (Fig. 2A). In this process, the acrylamide (AAm) solution was infused between the colloidal crystal template and the microgroove template to form a sandwich-like structure (Fig. S1). After ultraviolet-induced gelation, the sandwich-like structure was immersed in hydrofluoric acid to remove the template, followed by a methacrylated gelatin (GelMA) coating (Fig. 2B and C and Fig. S2). The obtained SCH inherited the characteristics of the colloidal crystal template, thereby displaying a unique structural color, while its characteristic reflection peak could be calculated via the Bragg–Snell equation, that is:

λ=2dsinθ (1)

where *d* and *θ* respectively refer to the lattice spacing and the glancing angle. When *d* remains constant, variations in the glancing angle *θ* have a discernible impact on the characteristic reflection spectrum (Fig. 2D and E). Furthermore, the mechanical properties of the SCH were tested and calculated (Fig. 2F and G and Fig. S3). It was found that 20% AAm presented less stretching and a greater tensile modulus, which was difficult to deform under the subtle cellular force, and lack of visual sensing monitoring function. In addition, 10% AAm with minor tensile modulus presented adequate extensibility and greater mechanical sensitivity but was fragile during the fabricating process. Particularly, 15% AAm exhibited robust mechanical strength, adequate extensibility, and exceptional sensitivity to subtle cellular forces. Hence, the SCH containing 15% AAm was chosen for the following experiment. These features granted the derived SCH sensitive mechanical responses, making it a promising candidate for visually assessing biomechanical features.

**Fig. 3.**
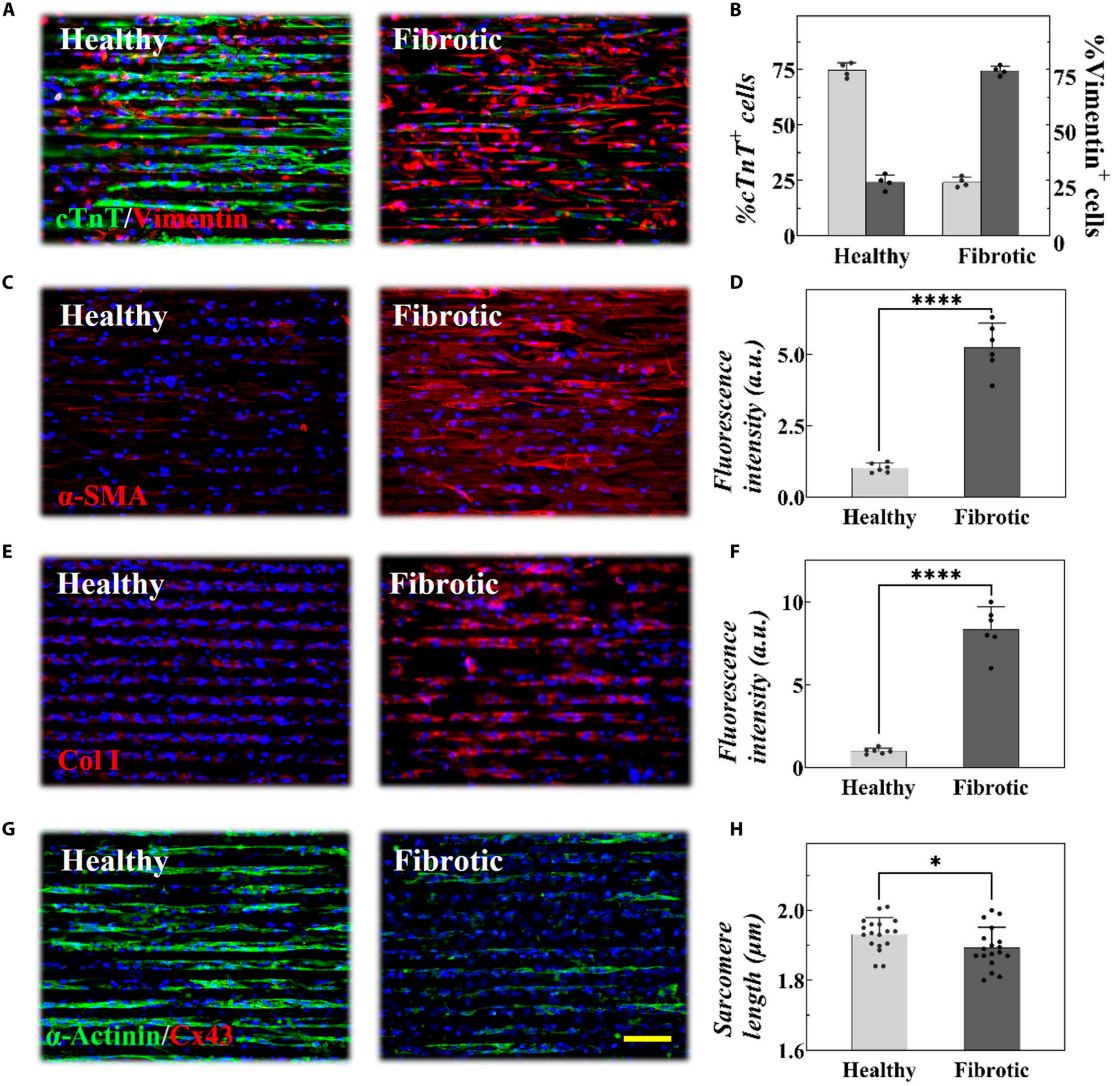
(A) Double-stained for cardiac troponin-T (cTnT) (cardiomyocyte marker) and vimentin (CFB marker) and counterstained with 4′,6-diamidino-2-phenylindole (DAPI). (B) Quantification of cellular composition. (C to F) Representative immunostaining images and plot of fluorescence intensity of healthy and fibrotic microtissue stained for α-smooth muscle actin (α-SMA) (C and D) and collagen I (Col I) (E and F). (G) Double-stained for α-actinin and Cx43 and counterstained with DAPI. (H) Corresponding sarcomere length measured from (G). Scale bar, 100 μm.

Playing a crucial role in tissue homeostasis, CFBs support angiogenesis and ECM maintenance while interacting with myocardial cells, blood vessels, and immune cells through paracrine activities [2,18,19]. Pathological fibrotic remodeling, stemming from CFB proliferation and ECM deposition, is implicated in nearly all cardiac diseases. To reproduce this remodeling in vitro, we designed healthy and fibrotic microtissue by planting the mixing of cardiomyocytes and CFBs at different ratios (healthy: cardiomyocytes to CFBs, 3:1; fibrotic: cardiomyocytes to CFBs, 1:3) on the SCHs [20,21]. The validation was performed through immunofluorescence staining (Fig. 3A and B). Particularly, benefiting from the surface microgrooves and the high biocompatibility of the SCHs, cardiomyocytes, and CFBs exhibited a guided cellular orientation consistent with that in vivo*,* an achievement unattainable on ordinary polystyrene dish (Figs. S4 and S5) [22]. After 4 days of culture, it was found that myofibroblasts and collagen content were marginally present in healthy microtissue but abundant in fibrotic microtissue (Fig. 3C to F). Interestingly, these collagen bundles appeared densely arranged in parallel within fibrotic microtissues, resembling those observed in in vivo fibrotic lesions. In addition, excessive ECM production reduced intercellular connectivity, while the marked disruption in connexin 43 (Cx43) gap-junction protein revealed lessened gap-junction formation in fibrotic microtissue (Fig. 3G and H). Referring to the above results, it could be concluded that such fibrotic microtissue could effectually reproduce compositional remodeling and collagen deposition of the fibrotic myocardium.

**Fig. 4.**
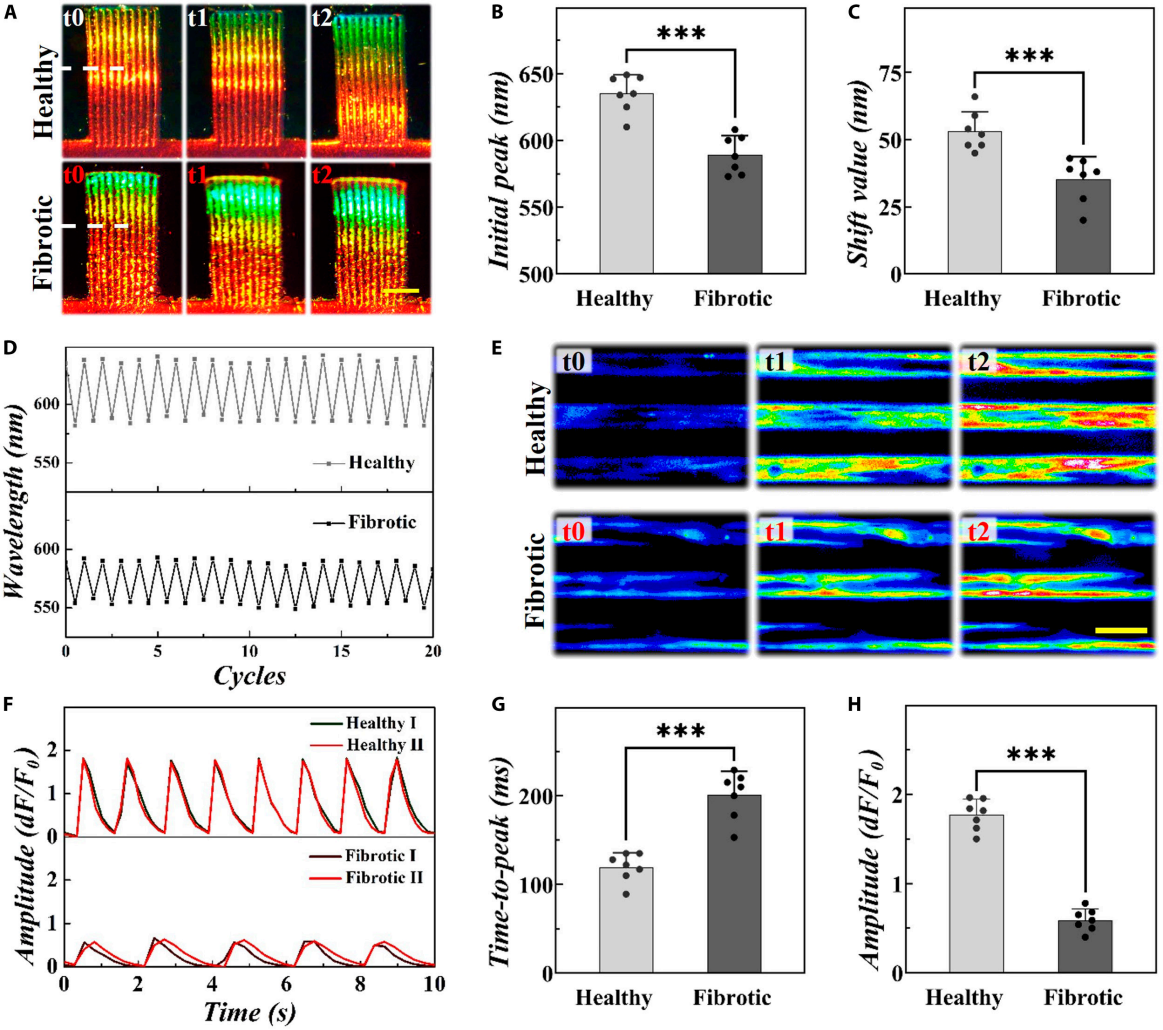
(A) Optical images of varying structural colors of the SCH in healthy and fibrotic microtissue during half myocardial cycle. (B) Initial peak at the marked position of the SCH in (A) during peak cardiomyocyte relaxation. (C) Maximum shift value at the marked position of the SCH in (A) during cardiomyocyte contraction. (D) Varying characteristic reflection peak at the marked position of the SCH in (A) during 20 myocardial cycles. (E to H) Calcium mapping (E), calcium transient traces (F), time-to-peak (G), and calcium flux amplitude (d*F*/*F*_0_) (H) of diverse microtissues. Scale bars, 250 μm (A) and 40 μm (E).

Moreover, we conducted multiparametric assessments to comprehensively explore the attributes of biomimetic fibrotic microtissue. Leveraging their exceptional biocompatibility and distinctive optical characteristics, SCHs demonstrated the ability to undergo simultaneous bending in response to cardiac forces, accompanied by discernible color changes and corresponding characteristic reflection peaks. Notably, the initial peak during peak cardiomyocyte relaxation indicated passive tension, with a smaller initial peak suggesting an increase in passive tension. In addition, the maximum shift value observed during cardiomyocyte contraction represented active contractile force, with decreasing shift value indicating a weaker contractile force. To illustrate, the optical images and reflection spectra were systematically detected in real time (Fig. 4A). It was found that the fibrotic microtissue presented markedly increased passive tension and diminished active contractile force, aligning with the impaired function commonly observed in clinic fibrotic heart (Fig. 4B to D). In addition, abnormal unsynchronized calcium waves as well as lower calcium transient amplitudes emerged in fibrotic microtissue (Fig. 4E to H and Movies S1 and S2). In particular, a longer time-to-peak period was observed in fibrotic microtissue. Therefore, this biomimetic fibrotic microtissue effectively emulated essential cardiac fibrosis features, including impaired contractile force, and aberrant calcium handling, providing a comprehensive evaluation of fibrotic characteristics and establishing the research foundation for the development of intelligent cardiac chips.

**Fig. 5.**
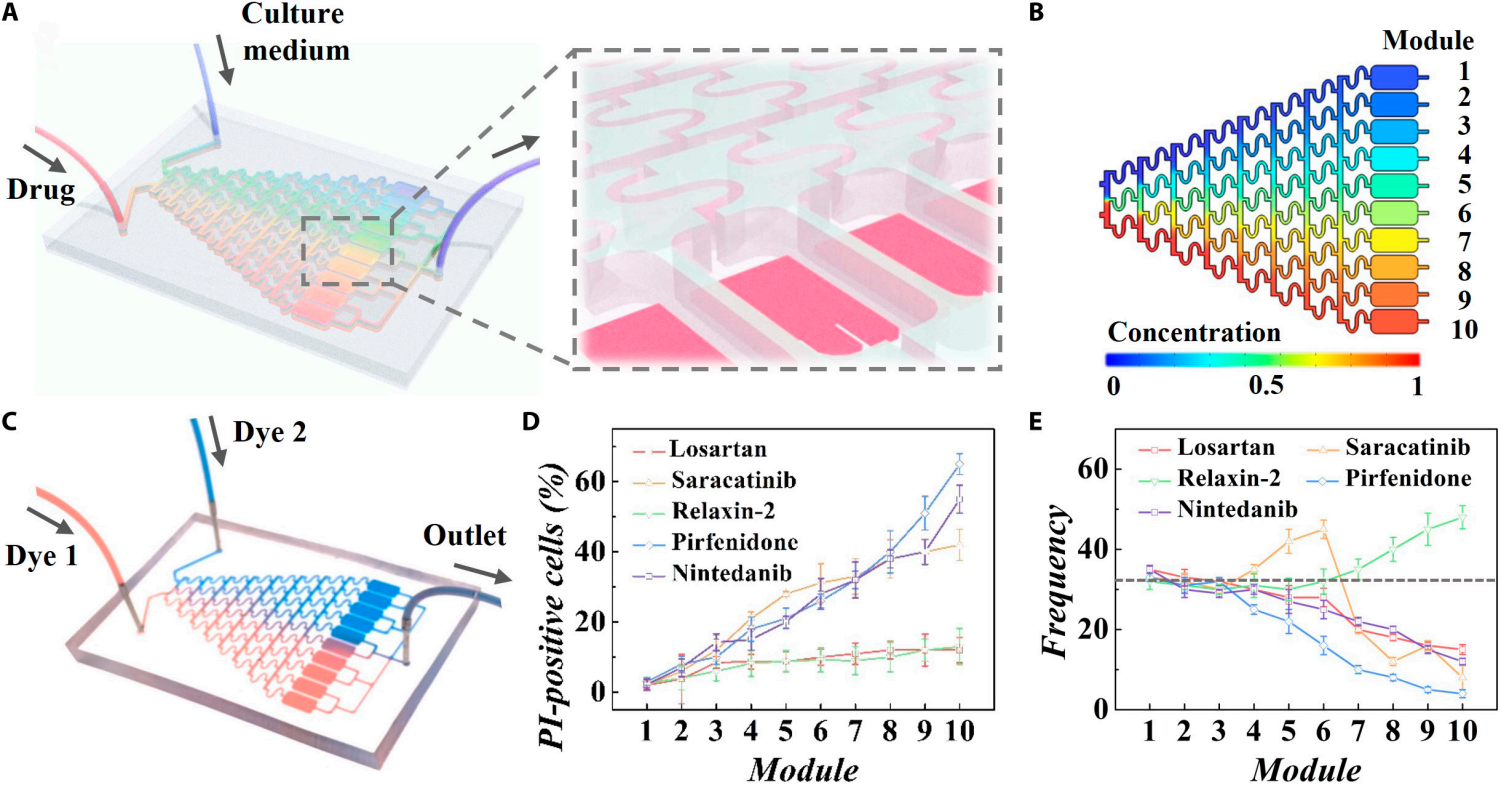
(A) Scheme of the cardiac fibrosis-on-a-chip system containing biomimetic microtissues. (B and C) Concentration gradient simulation (B) and optical image (C) of the system. (D and E) Quantification of percentage of dead cells (D) and cell beating frequency (beats/min) (E) in different modules.

To implement high-throughput drug screening, we integrated the biomimetic fibrotic microtissues in microfluidic chips to acquire cardiac fibrosis-on-a-chip systems (Fig. 5A). Microfluidic chips, widely embraced for biomedical research, serve as potent tools for recapitulating in vivo microenvironments [23–26]. By integrating diverse concentration gradient generation (CGG) networks, these chips can precisely regulate spatiotemporal fluid distribution, facilitating the creation of a tailored microenvironment with specific physical and chemical parameters [27–30]. This capability enables the establishment of high-throughput drug screening systems [31,32]. The utilization of classic “Christmas tree” models for constructing the CGG network, designed to generate 10 drug concentrations for each drug, exemplifies this approach [33,34]. The drug response module comprised individual microchambers incorporating biomimetic fibrotic microtissue, enabling cellular interactions with distinct drug concentrations without cross-interference. To find the appropriate inlet velocity and ensure proper diffusion of molecules applied from the customized “Christmas tree” channels, a simulation of the drug diffusion proceeded with COMSOL Multiphysics via the Navier–Stokes equation [35]. Simulation results demonstrated that fluids achieved complete mixing and transitioned to the next channels at an inlet velocity of 10^−6^ m/s, while an excellent drug concentration gradient was formed from module 1 to module 10 (Fig. 5B, Fig. S6, and Movie S3). After optimization, 2 dyes were pumped into the inlets, resulting in the observation of color mixture distribution across the channels (Fig. 5C). It was observed that the dyes in the drug response module presented a gradient distribution consistent with the theoretical prediction.

To establish a safe range of compound dosages for drug efficacy screening, a preliminary investigation into cell viability and arrhythmogenic response was conducted, aligning with the manufacturer’s instruction and pertinent research. In this study, 5 anti-fibrotic drugs were introduced into the drug inlet of the microfluidic chip to establish distinct drug concentration gradients. The drugs administered included losartan (20 μM, Ang-II receptor blocker), saracatinib (5 μM, kinase inhibitor), relaxin-2 (200 ng/ml, peptide hormone), pirfenidone (10 mM, TGF-β inhibitor), and nintedanib (10 μM, TGF-β inhibitor). Quantitative results revealed that elevated doses of pirfenidone, saracatinib, and nintedanib induced notable cell death and abnormal myocardial beating in fibrotic microtissues (Fig. 5D and E). Conversely, higher doses of losartan and relaxin-2 led to abnormal beating, with cell viability remaining unaffected. After comprehensive consideration, optimal drug response modules were selected for subsequent drug efficacy assessments, with losartan to module 6, saracatinib to module 3, relaxin-2 to module 6, pirfenidone assigned to module 3, and nintedanib to module 4. Later, live/dead cell assay and cell proliferation experiments were conducted at the optimal drug concentrations. The results demonstrated that the selected drug concentrations exhibited no detrimental effects on cellular viability (Fig. S7). Additionally, a subtle impact on cell proliferation was observed with saracatinib and pirfenidone (Fig. S8).

**Fig. 6.**
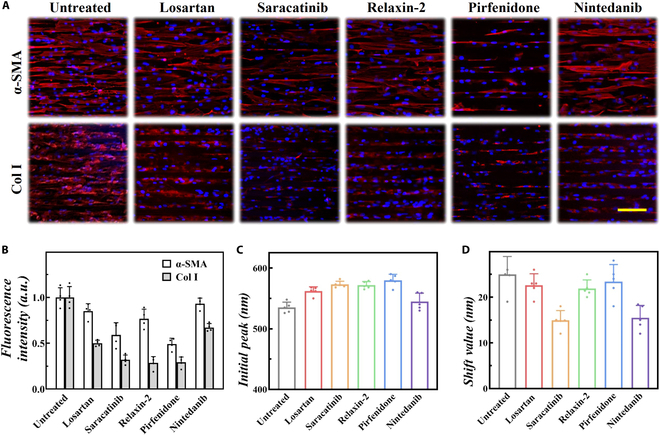
Anti-fibrotic drug efficacy under treatment. (A and B) Representative immunofluorescence images (A) and plot of fluorescence intensity (B) in fibrotic microtissues with or without anti-fibrotic treatments. (C and D) Initial peak (C) and shift value (D) of the SCH during multiple myocardial cycles. Scale bar, 100 μm (A).

To comprehensively evaluate drug efficacy on pathological remodeling, fibrotic microtissues were characterized after undergoing 4-day drug treatment, including CFB activation and collagen synthesis (Fig. 6A and B). Compared to the untreated microtissue, it was found that saracatinib and pirfenidone partly reduced the expression of α-smooth muscle actin (α-SMA), but losartan, relaxin-2, and nintedanib had inadequate effects. Besides, all 5 drugs demonstrated efficacy in reducing collagen deposition throughout the treatment period, especially saracatinib, relaxin-2, and pirfenidone. On this basis, the optical images and reflection spectra were systematically detected at the treatment endpoint. It was found that the drug-treated microtissue presented markedly different spectra patterns and structural color distributions (Figs. S9 and S10). Specifically, all 5 drugs demonstrated an increased initial peak, although the shift value presented no marked change and even decreased (Fig. 6C and D). The increased initial peak represented a reduced passive tension, demonstrating an improved cardiac diastolic function due to a weakened CFB activation and decreased collagen deposition. Compared to nintedanib, a greater reduction in passive tension was observed in the other 4 drug treatment groups, demonstrating a correlation with the levels of α-SMA expression. However, the shift value results indicated that there was no obvious improvement in active contractile force and even a reduction. Taken together, nintedanib exhibited limited efficacy in reducing fibrosis, whereas pirfenidone showcased promise in mitigating maladaptive passive tension. Thus, such biomimetic fibrotic chips hold substantial potential for advancing high-throughput drug screening applications.

## Conclusion

In conclusion, we have presented a novel cardiac fibrosis-on-a-chip based on SCHs for optical high-throughput drug screening. With the assistance of the surface-topological SCHs, the assembled fibrotic module presented densely packed collagen bundles arranged in parallel configurations, mimicking in vivo fibrotic lesions. The cardiac contraction and relaxation-induced synchronous deformation of SCHs leaded to a discernible shift in the structural color and characteristic reflection spectrum. Such design lays the foundation for a comprehensive visual criteria framework, providing an effective and reliable testing platform for subsequent anti-fibrotic drug screening. Based on these features, we integrated the fibrotic microtissue into a microfluidic gradient chip, and the obtained high-throughput drug screening chip achieved exceptional accuracy and efficiency in screening various promising anti-fibrotic drugs. The obtained results suggest that our high-throughput chip system holds great potential as an effective tool for evaluating efficacy and establishing dosage standards for newly developed drugs.

Anti-fibrotic drug screening traditionally utilized high-density microplates to rapidly identify candidates with anticipative therapeutic effects from extensive chemical libraries. Such plates necessitate sophisticated and costly automated liquid handling systems and microscopy equipment for assay analysis. However, these resources are often unavailable in conventional laboratories. In contrast, our microfluidic system employed advanced liquid manipulation and compartmentalization, enabling precise spatiotemporal control of drug distribution. Moreover, conventional methods for measuring cardiac force often rely on intricate equipment and involve complex analysis procedures, resulting in both cost and time constraints. In contrast, our system introduced SCHs as a novel approach to gauge cardiac force. SCHs manifested real-time optical signals visible to the naked eye, presenting a straightforward and economical method for rapidly screening various anti-fibrotic drugs. The utilization of these structural color materials within a microfluidic gradient chip holds the potential to revolutionize precision medicine technology and establish a streamlined strategy for preclinical research.

## Methods

### Materials

Gelatin, pancreatin, trypsin, AAm, methacrylic anhydride, photo-initiator 2-hydroxy-2-methylpropiophenone (HMPP), losartan, pirfenidone, and nintedanib were purchased from Sigma-Aldrich. Calcein-AM/propidium iodide (PI) double stain kit was supplied by Beyotime. Collagenase type 2 and Fluo-4 Direct Calcium Assay Kit were acquired from Worthington and Invitrogen, respectively. 4′,6-Diamidino-2-phenylindole (DAPI), relaxin-2, and antibodies were supplied by Abcam. Saracatinib was purchased from Selleck Chemicals LLC. Alexa Fluor 488 phalloidin, the secondary antibodies, fetal bovine serum, Dulbecco’s modified Eagle’s medium/nutrient mixture F-12, and Hanks’ balanced salt solution were supplied by Life Technologies. Polydimethylsiloxane (PDMS) was acquired from Dow Corning Corporation.

### Preparation of SCHs

The colloidal crystal template was assembled by monodisperse silica nanoparticles based on the vertical deposition method as in our previous report [36]. The microgroove template with 20-μm spacing was fabricated by replicating the silicon wafer. The AAm solution was injected between the microgroove template and the colloidal crystal template. In addition, the solution composed of 5 wt% GelMA (monomers) and 0.5 vol% HMPP (photoinitiator) was prepared. Then, the obtained sandwich-like structure was gelated by ultraviolet and immersed in hydrofluoric acid to remove the template, followed by a GelMA coating. Finally, the SCHs were soaked in deionized water to eliminate any unreacted chemicals.

### Extraction of primary cells

Cardiac cells were extracted from neonatal Sprague–Dawley rats and subjected to cutting and digesting procedures to attain a cardiac cell suspension. Following centrifugation, the resuspended cardiac cells were added to a 6-well plate. Finally, cardiomyocytes and CFBs were separated by using the differential adhesion method.

### Immunofluorescence and image analysis

Cells were fixed utilizing a 4% paraformaldehyde solution, followed by permeabilization with a 0.25% Triton X-100 solution, and subsequent blocking with 5% bovine serum albumin. F-actin staining was achieved using Alexa Fluor 488 phalloidin. Immunostaining was performed using specific antibodies, including rabbit anti-Col I (collagen I), rabbit anti-vimentin, rabbit anti-α-SMA, rabbit anti-α-actinin, mouse anti-cTnT (cardiac troponin-T), and goat anti-Cx43 with the secondary antibodies goat anti-rabbit-Alexa Fluor 488, goat anti-rabbit-Alexa Fluor 647, donkey anti-goat-Alexa Fluor 647, and goat anti-mouse-Alexa Fluor 488. Following staining, samples were stained with DAPI. Images were captured employing confocal microscopy (Zeiss). The analysis of fluorescence intensity was conducted employing ImageJ.

### Fabrication of microfluidic chip

To obtain a microfluidic chip, PDMS and the curing agent, combined in a ratio of 9:1, underwent meticulous mixing and evacuation to ensure uniformity. Subsequently, the resulting PDMS mixture was utilized for replicating customized polymethylmethacrylate templates featuring distinctive “Christmas tree” channels and individual drug response modules. Following this, SCHs were incorporated into the individual modules. Lastly, following plasma treatment and ultraviolet sterilization, the integrated microfluidic chip was prepared for the following experiment.

### Cell viability analysis

Cell viability was assessed using Cell counting kit-8 (CCK-8, Beyotime). Specifically, cardiac cells were cultured on the SCHs with various drug treatments. During measurement, the medium was substituted with CCK-8 medium and incubated for 2 h at 37 °C. Finally, the absorbance at 450 nm was quantified employing a microplate reader (Tecan Infinite M Nano, Switzerland).

### Live/dead cell assay

Calcein-AM/PI double stain kit was used to stain cardiac cells in different modules according to the instructions by the manufacturer. Cardiac cells were imaged with a fluorescence microscope (Leica).

### Calcium imaging

Fluo-4 Direct Calcium Assay Kit was employed for calcium activity assessment following the manufacturer’s instructions. Calcium images were acquired by 2-photon microscopy (Zeiss).

### Characterization

The microstructures during SCH fabrication were characterized by scanning electron microscopy (SEM) (Hitachi). The mechanical properties were characterized by a tensile testing machine (INSTRON). Optical images and reflection spectra were characterized by an optical microscope (Olympus) and an angle-resolved resolution spectrometer (Idea Optics), respectively.

## Data Availability

The authors confirm that the data supporting the findings of this study are available within the article and/or its Supplementary Materials.
