# Supplementary material for: A Biomimetic Optical Cardiac Fibrosis-on-a-Chip for High-Throughput Anti-Fibrotic Drug Screening
Source: Research (Wash D C). 2024 Sep 12;7:0471. doi: 10.34133/research.0471 (PMC11391215; doi:10.34133/research.0471)
Supplement: Supplementary 1 — Figs. S1 to S10 Movies S1 to S3 [file research.0471.f1.zip › Supplemental Information.docx]

Supplemental Information

**A biomimetic optical cardiac fibrosis-on-a-chip for high-throughput anti-fibrotic drug screening**

Yixuan Shang^1^, Dongyu Xu^2^, Lingyu Sun^1, 2,^ *, Yuanjin Zhao^1,2,^*, Lingyun Sun^1,3,^*

^1^ Department of Rheumatology and Immunology, Nanjing Drum Tower Hospital, Affiliated Hospital of Medical School, Nanjing University, Nanjing 210008, China

^2^ State Key Laboratory of Bioelectronics, School of Biological Science and Medical Engineering, Southeast University, Nanjing 210096, China

^3^ Department of Rheumatology and Immunology, The First Affiliated Hospital of Anhui Medical University, 218 Jixi Road, Hefei 230022, China

E-mail: 1035496126@qq.com; yjzhao@seu.edu.cn; lingyunsun@nju.edu.cn

**
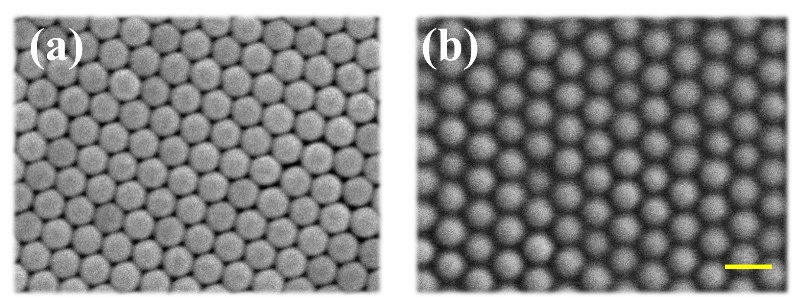
**

**Fig. S1.** SEM image of the colloidal crystal template (a) and the colloidal crystal template filled with gelated AAm hydrogel (b). The scale bars are 0.5 μm.


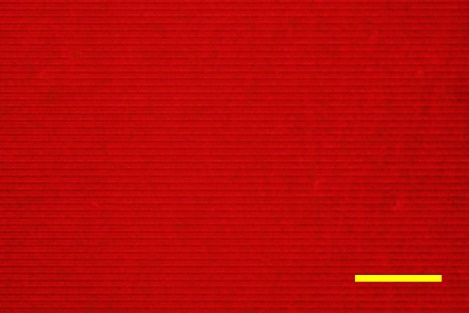


**Fig. S2.** Optical image of the SCH. The scale bar is 500 μm.

**
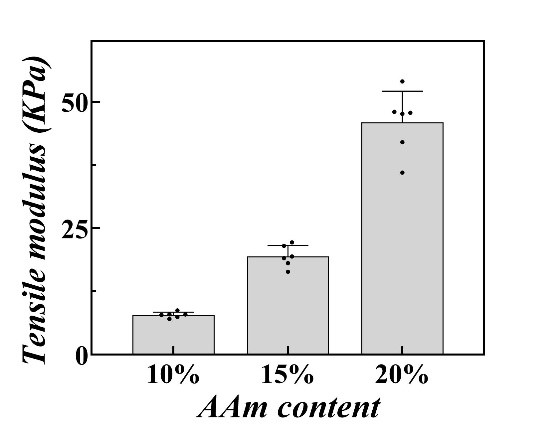
**

**Fig. S3.** Tensile modulus of the SCHs with diverse AAm concentrations.

**
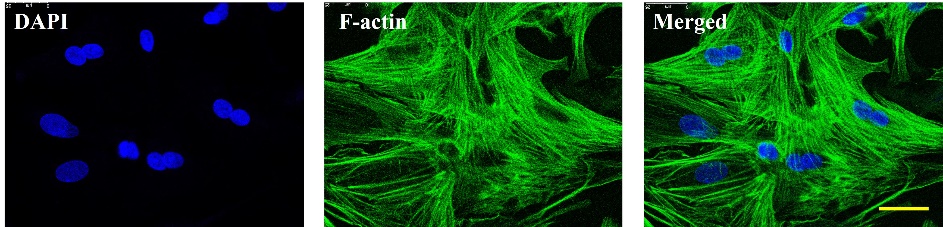
**

**Fig. S4.** Fluorescence images of cardiomyocytes cultured on the ordinary polystyrene dish. The scale bar is 20 μm.


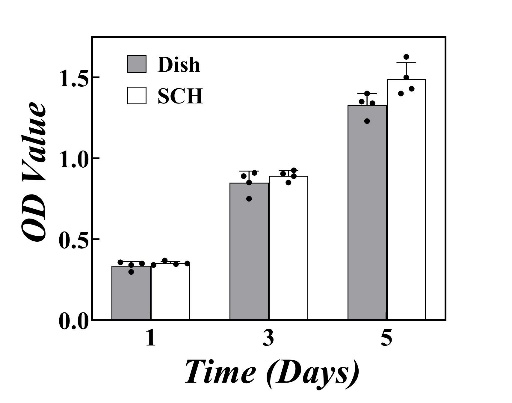


**Fig. S5.** Viability testing of CFBs cultured on ordinary polystyrene dishes and SCHs for 1 day, 3 days, and 5 days, respectively.


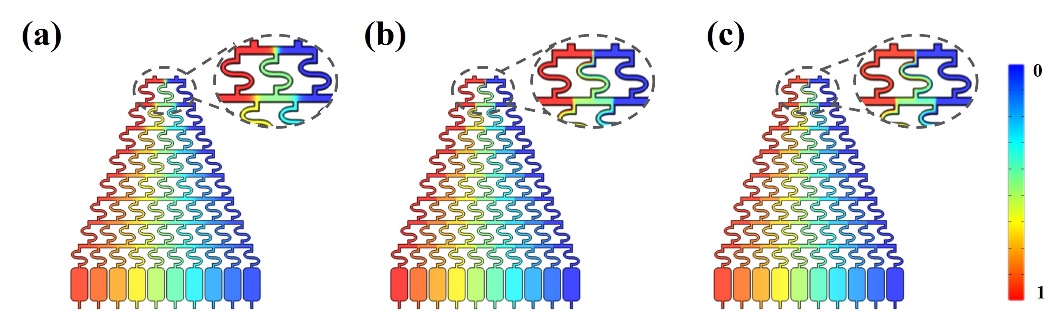


**Fig. S6.** Simulation results at different speeds. (a)10^-6^ m/s, (b)10^-4^ m/s, (c) 10^-2^ m/s.

**
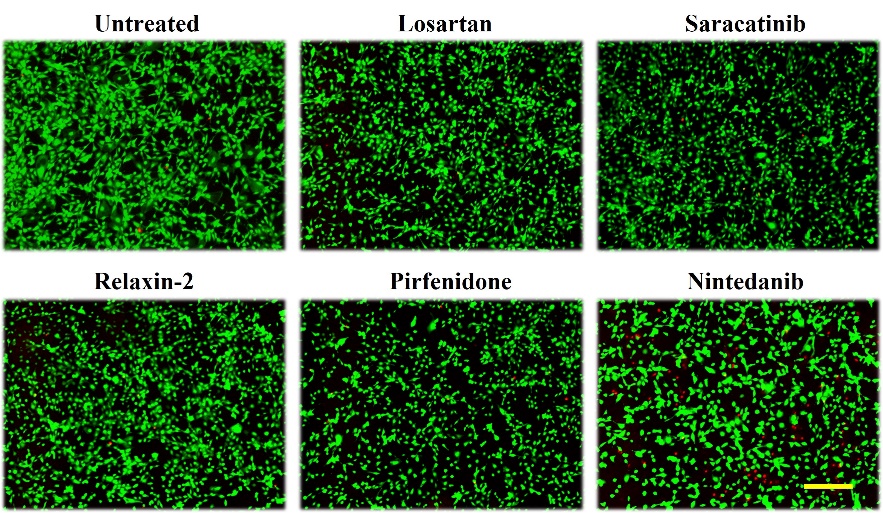
**

**Fig. S7.** Representative images showing cardiac cell viability under anti-fibrosis drug treatments on Day 4. Green represents live cells labeled with calcein and red represents dead cells labeled with PI. The scale bar is 300 μm.


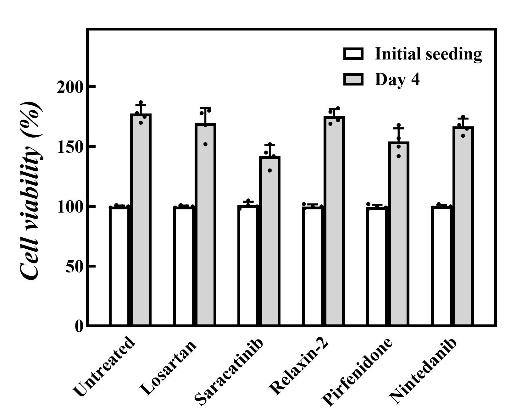


**Fig. S8.** Measurement of cell proliferation under anti-fibrosis drug treatment.

**
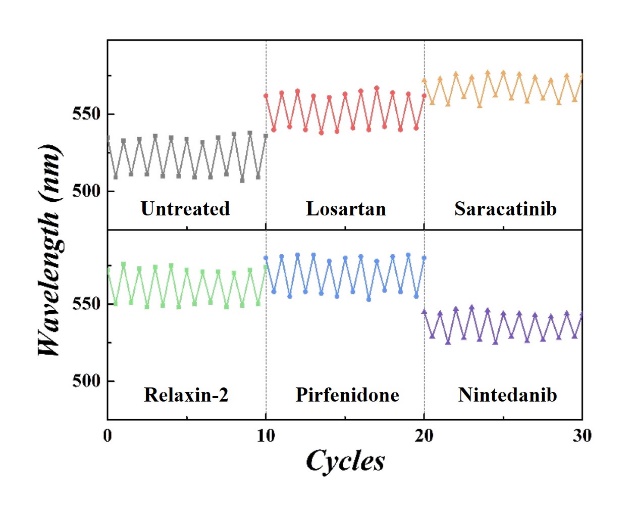
**

**Fig. S9.** Characteristic reflection peak on the fibrotic microtissues during ten myocardial cycles.


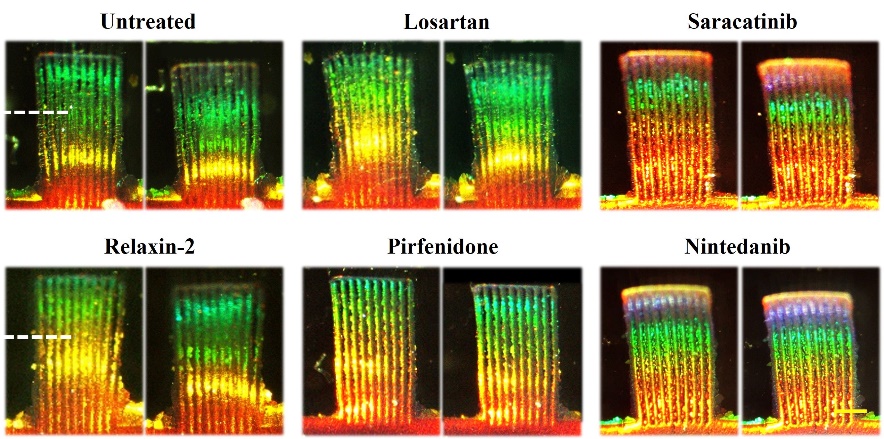


**Fig. S10.** Optical images of fibrotic microtissues during peak cardiomyocyte relaxation (left) and contraction (right). The scale bar is 200 μm.

**Description of Supporting Movie S1 to Movie S3：**

**Movie S1:** Calcium mapping in a healthy microtissue.

**Movie S2:** Calcium mapping in a fibrotic microtissue.

**Movie S3:** COMSOL simulation of the drug diffusion.
