# Supplementary figures and images for: A Biomimetic Optical Cardiac Fibrosis-on-a-Chip for High-Throughput Anti-Fibrotic Drug Screening
Source: Research (Wash D C). 2024 Sep 12;7:0471. doi: 10.34133/research.0471 (PMC11391215; doi:10.34133/research.0471)

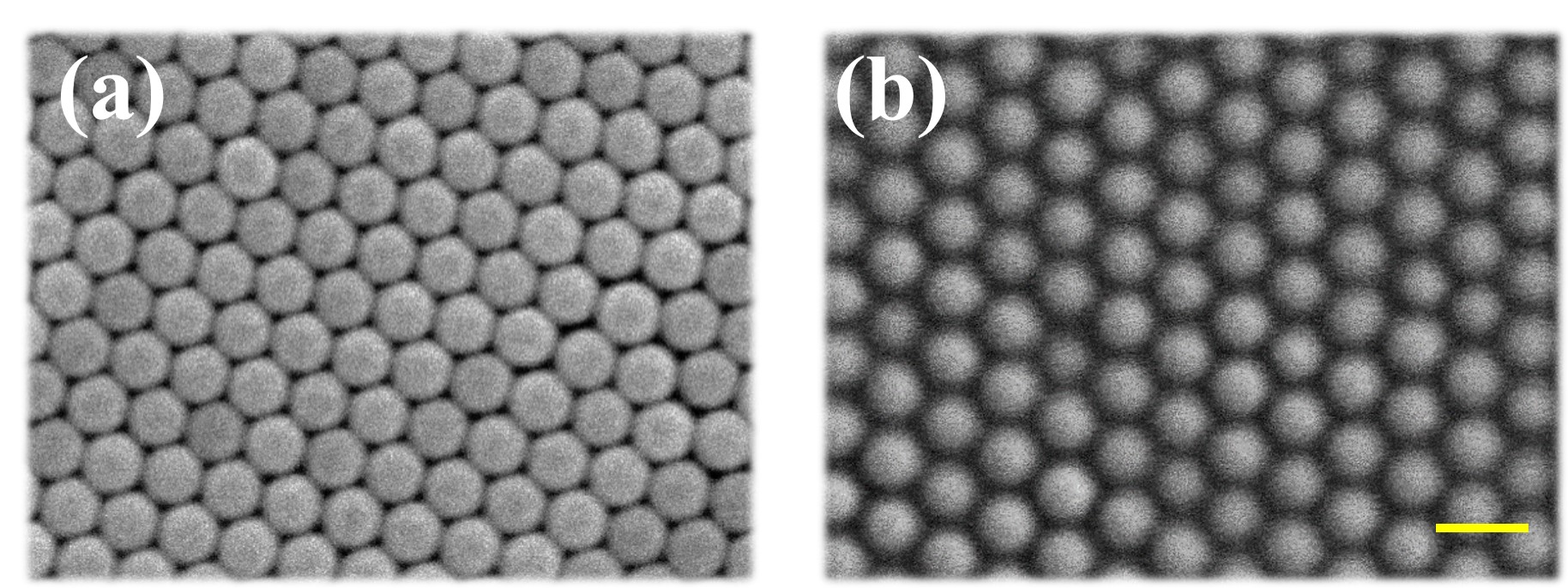

Supplement: Supplementary 1 — Figs. S1 to S10 Movies S1 to S3 [file research.0471.f1.zip › Figure S1.jpg]

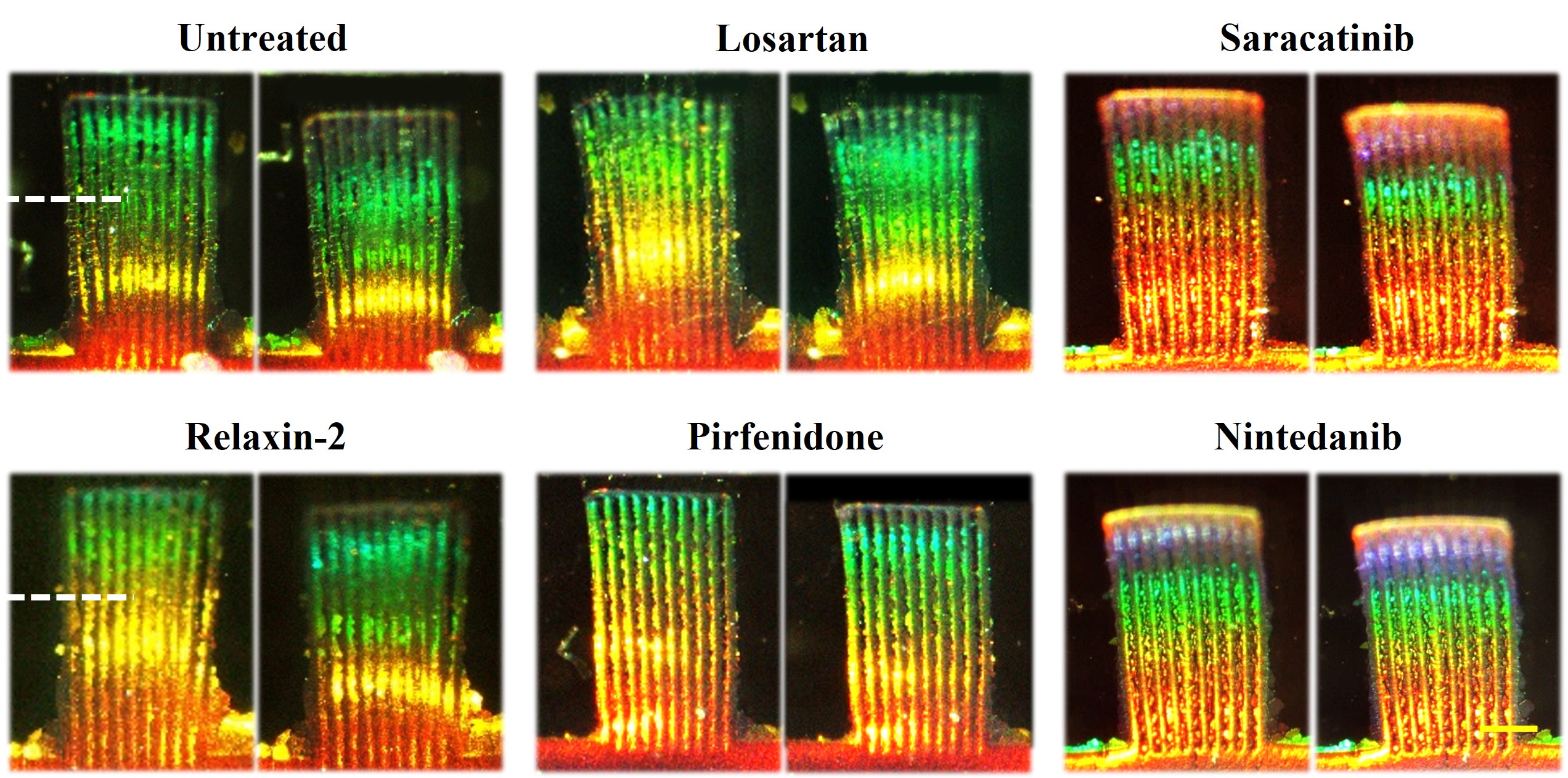

Supplement: Supplementary 1 — Figs. S1 to S10 Movies S1 to S3 [file research.0471.f1.zip › Figure S10.jpg]

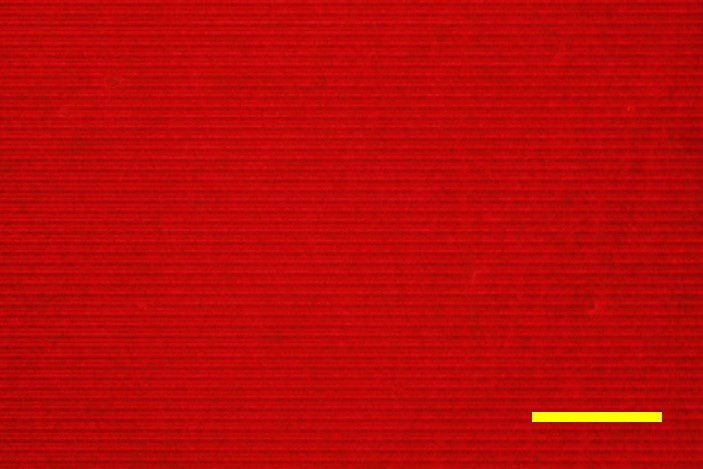

Supplement: Supplementary 1 — Figs. S1 to S10 Movies S1 to S3 [file research.0471.f1.zip › Figure S2.jpg]

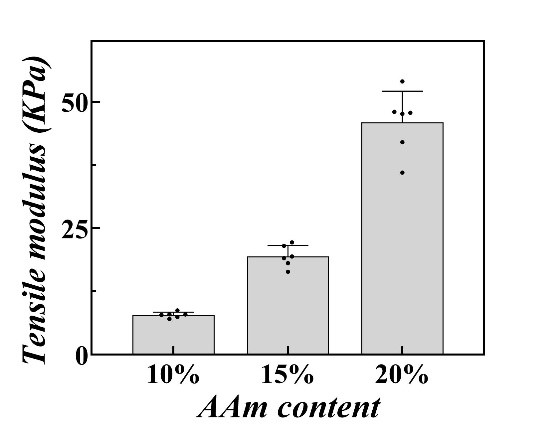

Supplement: Supplementary 1 — Figs. S1 to S10 Movies S1 to S3 [file research.0471.f1.zip › Figure S3.jpg]

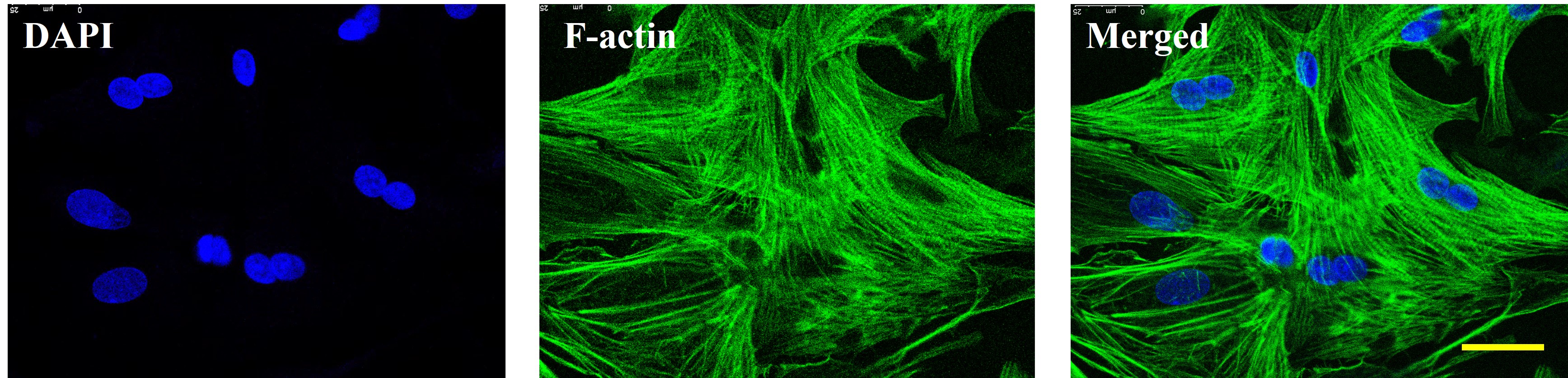

Supplement: Supplementary 1 — Figs. S1 to S10 Movies S1 to S3 [file research.0471.f1.zip › Figure S4.jpg]

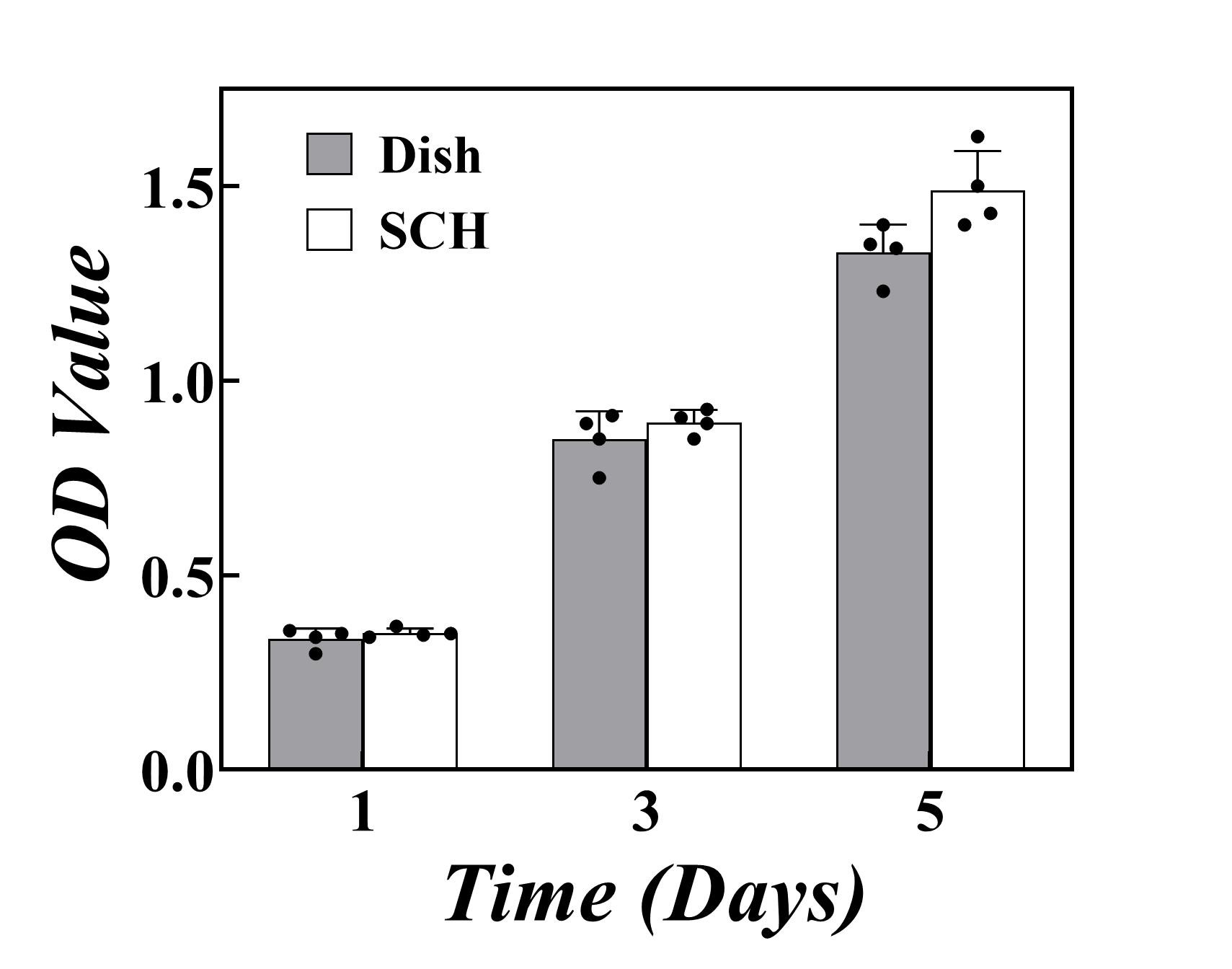

Supplement: Supplementary 1 — Figs. S1 to S10 Movies S1 to S3 [file research.0471.f1.zip › Figure S5.jpg]

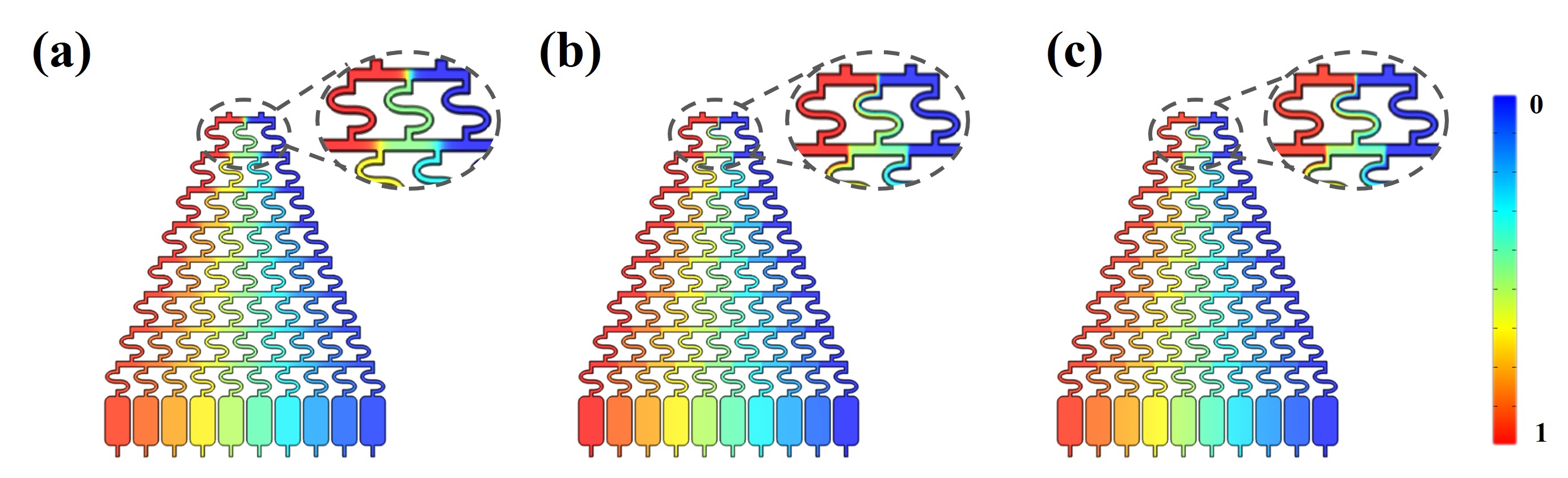

Supplement: Supplementary 1 — Figs. S1 to S10 Movies S1 to S3 [file research.0471.f1.zip › Figure S6.jpg]

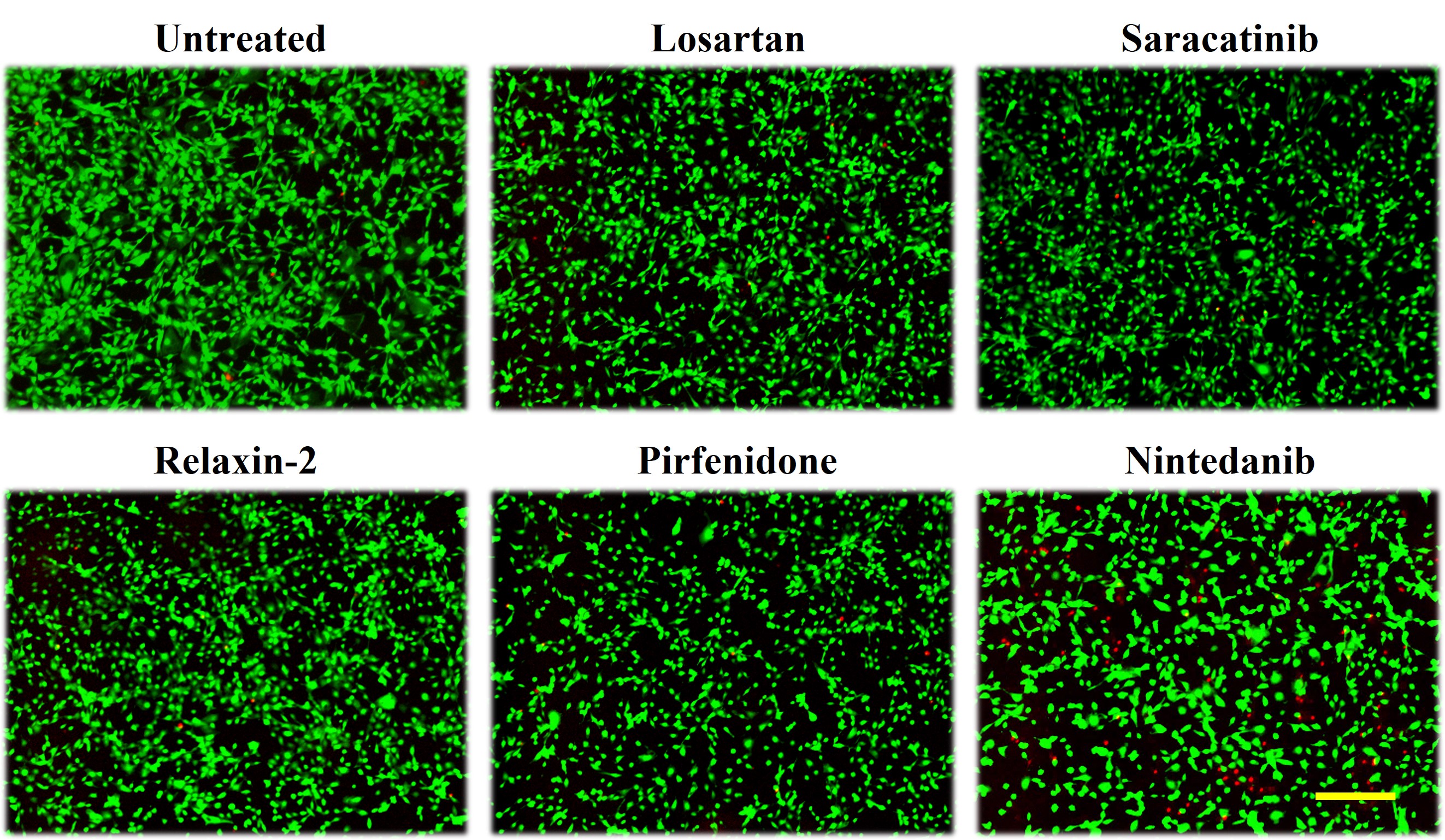

Supplement: Supplementary 1 — Figs. S1 to S10 Movies S1 to S3 [file research.0471.f1.zip › Figure S7.jpg]

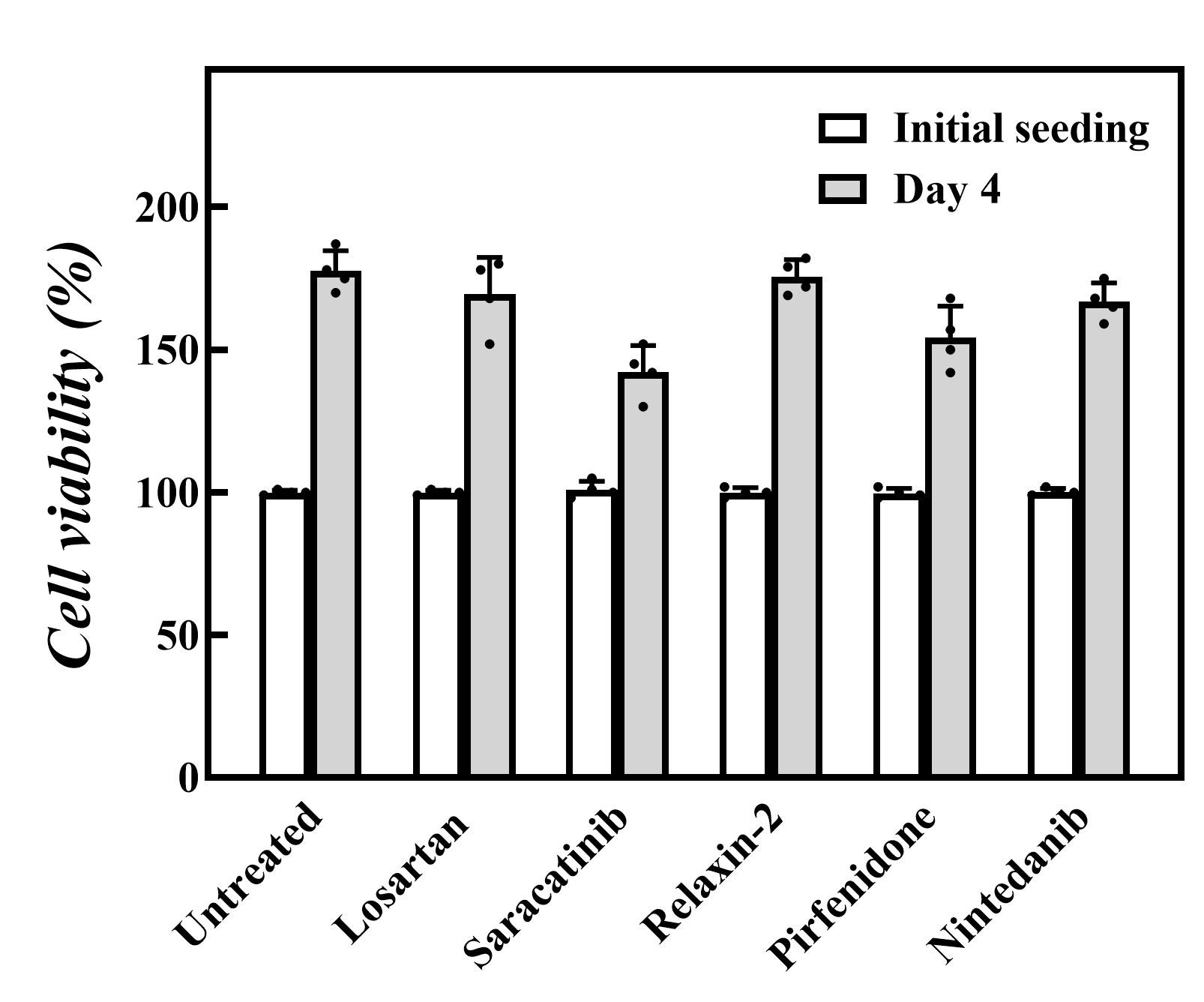

Supplement: Supplementary 1 — Figs. S1 to S10 Movies S1 to S3 [file research.0471.f1.zip › Figure S8.jpg]

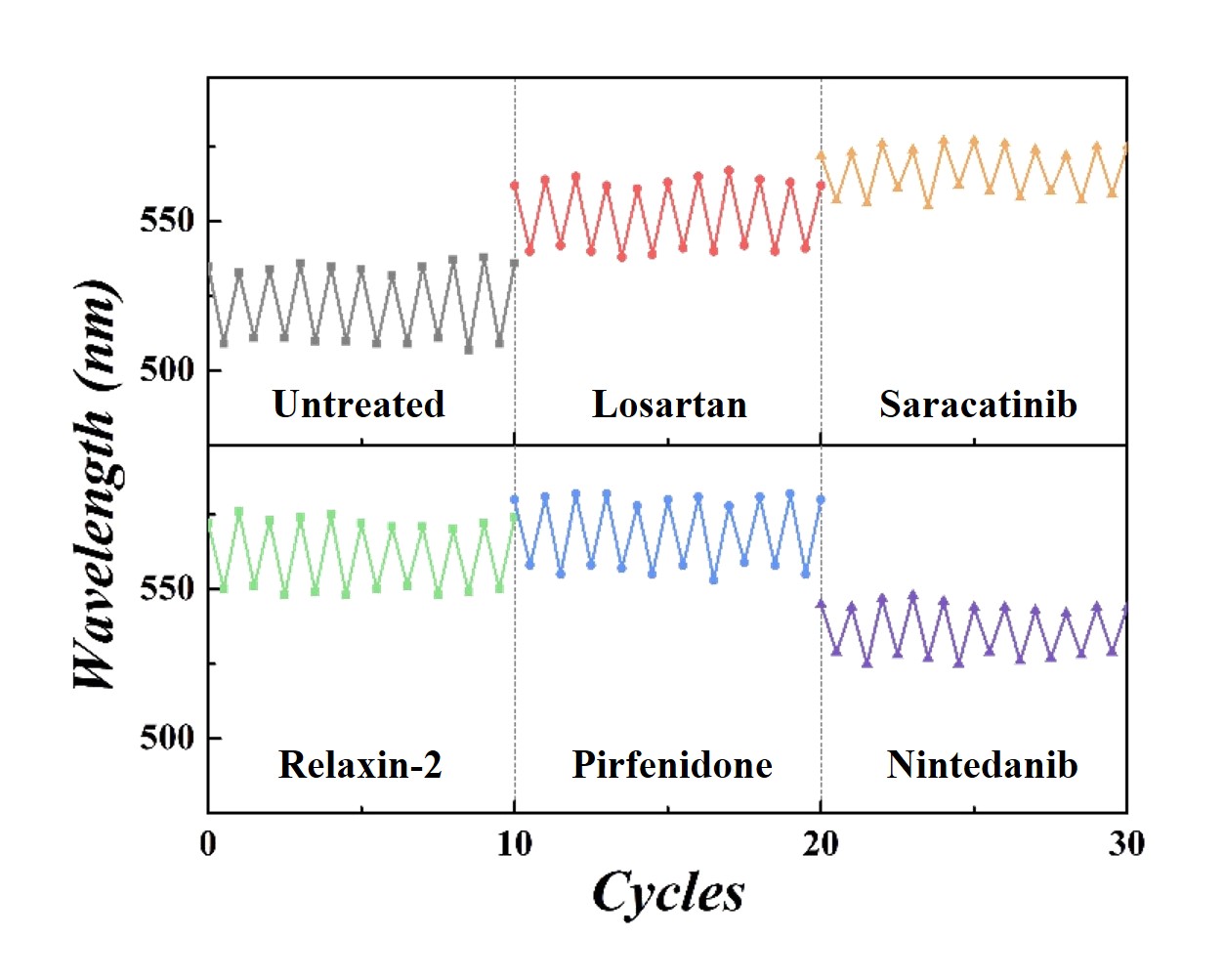

Supplement: Supplementary 1 — Figs. S1 to S10 Movies S1 to S3 [file research.0471.f1.zip › Figure S9.jpg]
